# Protocol for isolation of microbiota-derived membrane vesicles from mouse blood and colon

**DOI:** 10.1016/j.xpro.2023.102046

**Published:** 2023-01-19

**Authors:** Saskia F. Erttmann, Nelson O. Gekara

**Affiliations:** 1 Department of Molecular Biology, Umeå Centre for Microbial Research (UCMR), Umeå University, 90187 Umeå, Sweden; 2 Infection Oncology Unit, Institute of Clinical Molecular Biology, Christian-Albrechts University of Kiel, UKSH, Schwanenweg 20, 24105 Kiel, Germany; 3 Department of Molecular Biosciences, The Wenner-Gren Institute, Stockholm University, 106 91 Stockholm, Sweden; 4 Faculty of Medicine, Institute of Medical Microbiology and Hygiene, Medical Center, University of Freiburg, Hermann-Herder-Str. 11, 79104 Freiburg, Germany

**Keywords:** Cell Separation/Fractionation, Immunology, Microbiology, Model Organisms

## Abstract

Bacterial membrane vesicles have emerged as gadgets allowing remote communication between the microbiota and distal host organs. Here we describe a protocol for enriching vesicles from serum and colon that could widely be adapted for other tissues. We detail pre-clearing of serum or colon fluids using 0.2-μm syringe filters and their concentration by centrifugal filter devices. We also describe vesicle isolation with qEV size exclusion columns and finally the concentration of isolated vesicle fractions for downstream analyses.

For complete details on the use and execution of this protocol, please refer to *Erttmann* et al. (2022).^1^

## Highlights

• Collection of mouse serum and colon samples
• Concentration of samples using centrifugal filter devices
• Isolation of membrane vesicles using size exclusion columns
• Concentration of isolated vesicle fractions using spin columns

## Abstract (editor highlights)

Publisher’s note: Undertaking any experimental protocol requires adherence to local institutional guidelines for laboratory safety and ethics.

## Graphical abstract

**Figure:**
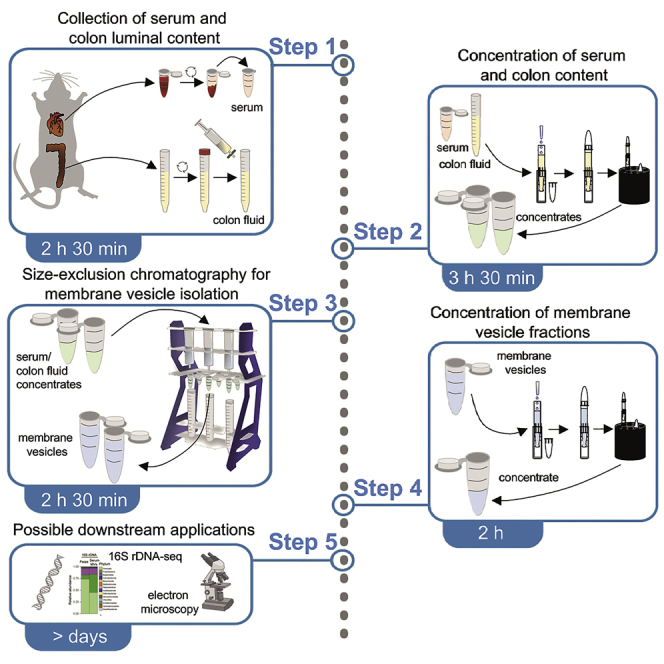

## Before you begin

### Institutional permissions

Animal breeding and experiments were carried out according to the guidelines set by the Umeå Regional Animal Ethic Committee (Umeå Regionala Djurförsöksetiska Nämnd), Approval no. A53-14 and A25-2019. *In vivo* experiments including the euthanasia of mice and collection of blood were performed at the UCCB. Mice were maintained under specific pathogen-free conditions. Age- and sex-matched, co-housed adult C57BL/6 mice (8–24 weeks) were used.

To conduct related experiments, permissions from the relevant institutions at the place of research are required.

### Material to prepare in advance

**Timing: 3 h**

1. To collect serum from mouse blood, prepare the following items.
  a. Clean several sets of forceps (stainless steel blunt-end forceps, Fisherbrand, Fisher Scientific, #11512123) and scissors (dissecting scissors, Fisherbrand, Fisher Scientific, #15207266) with dishwashing detergent, then rinse with an excess of ultrapure water for example from a Milli-Q IQ Water Purification System.
  b. Dry forceps and scissors, then place them into sterilizer pouches (Synergy Healthcare Azo Self Seal Sterilizer Pouches, 200 × 90 mm, Synergy Healthcare MBA1081, Fisher Scientific, #15601984).
  c. Sterilize the instruments using an appropriate autoclave and sterilization program (121°C, 20 min).
2. Prepare 1× Dulbecco’s phosphate buffered saline (DPBS) from powder (DPBS, powder, no calcium, no magnesium, Gibco, Thermo Fisher Scientific, #21600010).
  a. Dissolve the powdered medium in distilled water under gentle stirring and adjust to a final volume of 1 L.
  b. Process the medium into sterile containers by membrane filtration using a Nalgene vacuum filtration system with a filter pore size of 0.2 μm (Merck, #Z358207) using a positive-pressure system.

## Key resources table

**Table:** 

REAGENT or RESOURCE	SOURCE	IDENTIFIER
**Chemicals, peptides, and recombinant proteins**		

(DPBS, powder, no calcium, no magnesium)	Gibco, Thermo Fisher Scientific	#21600010
Benzonase nuclease (HC purity >99%) (if extravesicular DNA digestion is performed)	Novagen, Merck	#71206
Pierce BCA protein assay	Thermo Scientific	#23225

**Critical commercial assays**		

qEVoriginal/70 nm Gen 2 Column	IZON	#ICO-70, 1004105
Amicon Ultra-2 Centrifugal Filter Units (Centrifugal concentrator, 3 kDa MWCO, 2 mL sample volume)	Merck, Millipore	#UFL200324
AMPure XP beads for MV DNA isolation and purification (if DNA isolation for rDNA-seq is performed)	Beckman Coulter	#A63881

**Experimental models: Organisms/strains**		

C57BL/6J Maintained under specific pathogen-free conditions; age- and sex-matched (8–24 weeks), co-housed	The Jackson Laboratory	#:000664 RRID: IMSR_JAX:000664

**Other**		

Nalgene vacuum filtration system with a filter pore size of 0.2 μm	Merck	#Z358207
Sterilizer pouches (Synergy Healthcare Azo Self Seal Sterilizer Pouches, 200 × 90 mm)	Synergy Healthcare, Fisher Scientific	#15601984
Forceps (stainless steel blunt-end forceps)	Fisherbrand, Fisher Scientific	#11512123
Scissors (dissecting scissors)	Fisherbrand, Fisher Scientific	#15207266
Millex 13 mm PVDF .22 μm Sterile RUO	Merck	#SLGVR13SL
Millex 13 mm PVDF .45 μm Sterile RUO	Merck	#SLHVR13SL
sterile spatula (Corning single use spatula/spoon, flat end)	Merck	#CLS3007
Test tube shakers Genie Vortex Mixer Model: Vortex-Genie 2	Carl Roth, Scientific Industries	#P505.1
Sterile 1.5 mL low protein binding collection tube	Thermo Scientific	#90411
Pipette tips large opening 100–1000 μL, with filter, sterile	Carl Roth	#CHC6.1

## Step-by-step method details

### Collection of serum from mouse blood

**Timing: 2 h 30 min**

This step describes the separation of serum from 24 mice to acquire enough material for the isolation of bacterial membrane vesicles from blood. A starting volume of ∼11 mL full blood from 24 mice yields ∼5 mL of serum. This is performed on the bench top or in a biosafety cabinet, depending on the biosafety level of the samples.

1. To collect blood from mice, mice are euthanatized by cervical dislocation.
  a. For this, with one hand, grasp the mouse directly behind the ears and apply pressure on the neck.
  b. With the other hand, hold the base of the tail.
  c. To dislocate the spinal column from the skull, keep the head of the mouse stationary, then with a firm steady motion lift the tail slightly and pull.
  d. By using your fingertips, palpate the neck region to be sure that the spinal column is dislocated from the head.
2. Opening of the thoracic cavity to take blood from the heart.
  a. Place the mouse on its’ back.
  b. Use a paper towel soaked in 70% ethanol to quickly disinfect the front of the mouse from the neck to the belly region.
  c. Then grasp a small flap of skin at the end of the sternum.
  d. With sharp, sterile scissors, cut a hole in the skin and musculature at the diaphragm.
  e. Next, insert one blade of scissors into the opening at the position of the sternum.
  f. To open the thoracic cavity, cut through the rib cage and bend the ribs to the side to expose the heart.
  g. With a sterile filter tip (pipette tips large opening 100–1000 μL, with filter, sterile, Carl Roth, #CHC6.1) attached to a micropipette, puncture and insert into the atrium of the heart, and carefully draw blood from the heart while avoiding clogging the pipet tip. In case of pre-mature clotting, collect the clots alongside with the blood.
  h. Transfer the collected blood into a sterile 1.5 mL microcentrifuge tube at room temperature (RT; 20°C).
3. Serum separation from full blood.
  a. Mix the blood collected in step 2.h by inverting the tube 5-times and then incubate the collected blood at RT for 30 min.
  b. Separate the serum from blood cells/clot by centrifugation at 1,500 ×*g* and 4°C for 10 min.
  c. Carefully transfer the upper phase, the serum, to a new 1.5 mL microcentrifuge tube by avoiding touching the blood clot.
  d. To clear the serum further, centrifuge the serum again at 1,500 ×*g* and 4°C for 10 min and transfer the upper phase to a new 1.5 mL microcentrifuge.
  e. Store the serum on ice until further processing.

**CRITICAL:** Try to prevent any contamination of the serum with traces of blood cells/clots. Aim at avoiding erythrocyte lysis during the serum preparation by gentle pipetting using pipette tips with a large opening. Contaminations of that kind might interfere with subsequent steps.

### Collection of mouse colon luminal content for BMV isolation

**Timing: 2 h 30 min**

This step describes how to harvest colon luminal content for the isolation of bacterial membrane vesicles. For enough material, 8 mice per sample (16 mice for 2 samples) are required. This procedure can be performed alongside the ’collection of serum from mouse blood’ described in steps 1–3 above.

4. Euthanatize mice by cervical dislocation as described in step 1.
5. Open the peritoneal cavity to sample the colon and its luminal content as follows.
  a. Place the mouse on its’ back.
  b. Use a paper towel soaked in 70% ethanol to quickly disinfect the front of the mouse from the sternum to the anus.
  c. Then grasp a small flap of skin and make a ventral incision between sternum and anus using sharp, sterile scissors to cut a hole in the skin.
  d. Remove the pelt by tearing the skin in the transverse plane.
  e. Open the peritoneal cavity by carefully cutting the peritoneal membrane.
    ***Note:*** Be careful not to cut any inner organs.
  f. Grasp the pyloric sphincter and put the stomach together with the small intestine aside until reaching the cecum.
  g. Cut below the cecum. Then grasp the proximal colon and cut the rectum.
  h. Place the colon into a sterile Petri dish.
  i. By applying gentle pressure on the colon, carefully guide the luminal content to the end of the colon until it leaves the luminal cavity.
  j. Collect the colon luminal content with a sterile spatula (Merck, Corning single use spatula/spoon, flat end, #CLS3007) into a 50 mL reaction tube in ice-cold 10 mL DPBS.
  k. Store samples on ice until further processing.
6. Pre-clearing of BMV-containing supernatants from colon luminal content.
  a. Vortex the colon content for 30 s at ∼1500 rpm (speed position 6; Test tube shakers Genie Vortex Mixer Model: Vortex-Genie 2, Carl Roth, Scientific Industries, #P505.1).
  b. Pellet colon luminal content at 4,000 ×*g*, 4°C for 5 min, and transfer the supernatant to a new 15 mL reaction tube.
  c. Centrifuge supernatant at 4,000 ×*g*, 4°C for 10 min and transfer the supernatant to a new 15 mL reaction tube.
  d. Repeat step c by spinning the supernatant at 4,000 ×*g*, 4°C for 10 min and transferring it to a new 15 mL reaction tube.
  e. Finally, spin the supernatant at 6,000 ×*g*, 4°C for 10 min, then transfer it to a new 15 mL reaction tube.
  f. To remove further contaminants, filter the supernatant using a 0.45 μm syringe filter into a new 15 mL reaction tube.
  g. To eliminate any remaining bacteria, filter the supernatant using a 0.2 μm syringe filter into a new 15 mL reaction tube. This should yield a total colon fluid volume of ∼4 mL.
  h. Hold the colon content fluid on ice, while equilibrating filter devices (see step below).

### Concentration of serum and colon fluid samples

**Timing: 3 h 30 min**

This step describes the concentration of the collected mouse serum and colon fluid. This is necessary because separation columns for BMV isolation can hold a maximum of 500 μL of sample volume.

**Figure 1:**
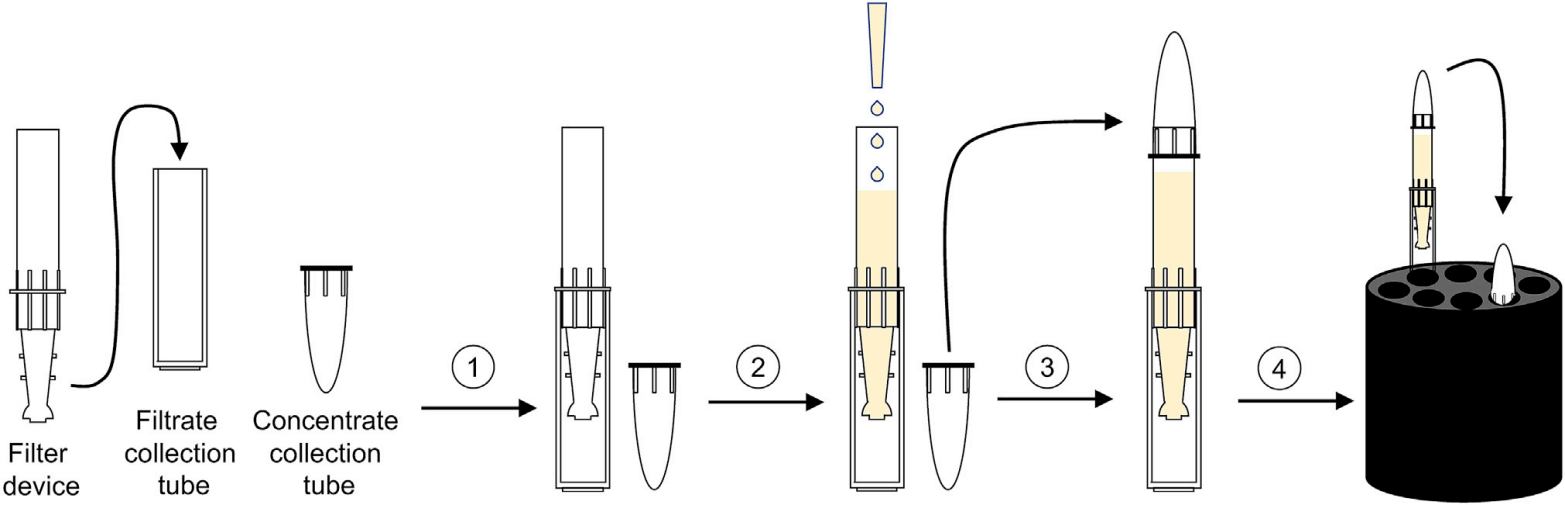
Schematic illustration of the assembly and loading of an Amicon Ultra-2 centrifugal filter device (1) Seal the filter device with the filtrate collection tube. (2) Load the sample into the filter device. (3) Add the concentrate collection tube on top of the filter device and push down to seal. (4) Insert the sealed filter device into the centrifuge rotor until the rim of the concentrate collection tube is inside the centrifuge rotor well.

**Figure 2:**
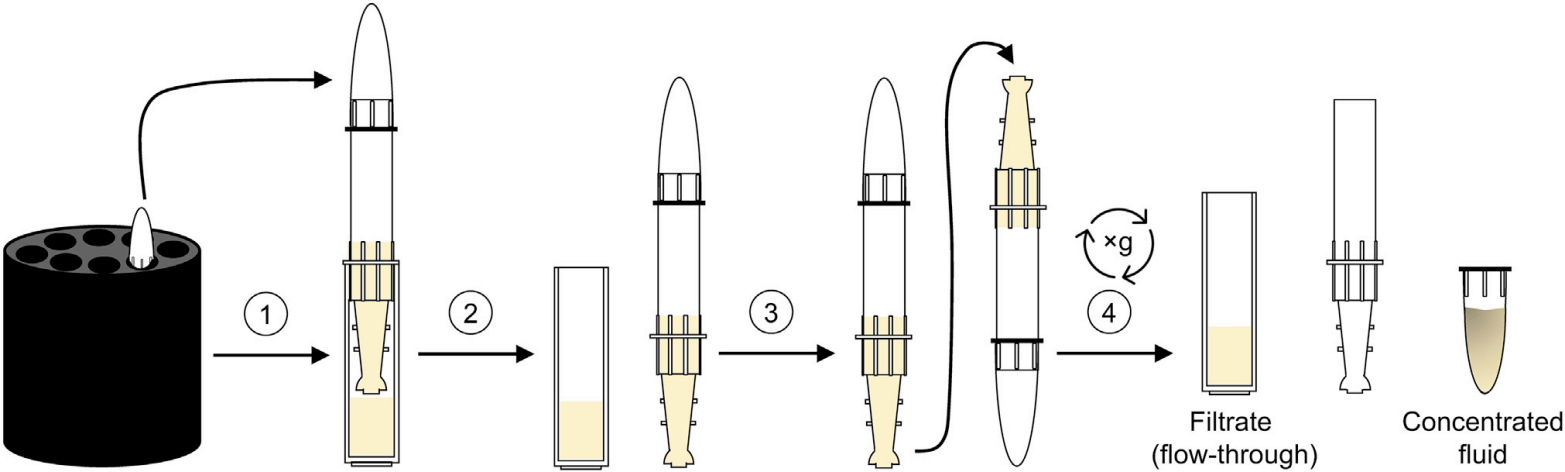
Schematics for the collection of concentrated serum and colon fluid from an Amicon Ultra-2 centrifugal filter device (1) Remove the whole filter device from the rotor. (2) Disconnect the filter device from the filtrate collection tube. (3) Invert the filter device with sealed concentrate collection tube. (4) Insert the inverted filter device with sealed concentrate collection tube into the centrifuge rotor and spin. Take the filter device with sealed concentrate collection tube out of the rotor and disconnect the concentrate collection tube containing the concentrated serum or colon fluid from the filter device.

7. To concentrate the collected serum and colon fluid to a final volume of 500 μL, use Amicon Ultra-2 centrifugal filter devices for volumes up to 2 mL with a cutoff of 3 kDa (Amicon Ultra 3K device – 3,000 NMW).
  a. Equilibrate each Amicon Ultra-2 centrifugal filter device with 500 μL ice-cold, sterile filtered DPBS to remove any traces of other buffers/chemicals contained in the filter devices used to preserve the resin**.**
    ***Note:*** The number of filter devices required depends on the volume and the viscosity of the sample.
    Equilibration of Amicon filters is performed as follows:
    i. Place the Amicon Ultra-2 filtrate devices into the filtrate collection tubes. Confirm that the filtrate devices are properly sealed in the collection tubes.
    ii. Load each filtrate device with 500 μL ice-cold, sterile filtered DPBS.
    iii. Cover the filtrate devices with the concentrate collection tubes by pushing the collection tubes onto the filtrate devices.
      ***Note:*** Be careful not to break the collection tubes. Appropriate sealing of all three device parts is required to prevent breaking of the device during centrifugation (see Figure 1 for schematic instructions how to seal the device parts).
    iv. Place the filter devices into the centrifuge with swinging buckets. Make sure the filter devices are in contact with the bottom of the rotor and that the rims of the concentrate collection tubes are inside the centrifuge rotor well. Counterbalance with similar devices if required.
    v. Centrifuge at 4,000 ×*g* and 4°C for 60 min.
      ***Note:*** The maximum speed when using a swinging bucket rotor is 4,000 ×*g*. If using a fixed angle rotor do not exceed 5,400 ×*g* when centrifuging viscous solutions such as serum to avoid breaking of filters.
    vi. Remove the filter devices from the centrifuge. Then disconnect the filter devices from the filtrate collection tubes containing the flow-through.
    vii. Discard the flow-through in the collection devices, then seal the filter devices again in the collection tubes.
  b. Use DPBS-equilibrated Amicon Ultra-2 centrifugal filter devices to load the serum and the colon fluid for subsequent concentration. Perform this as follows:
    i. Remove the concentrate collection tube from the filter device.
    ii. Load filtrate devices each with 1.5 mL ice-cold serum collected in steps 1–3 (*Preparation of serum from mouse blood*) or with 1.5 mL ice-cold colon fluid collected in steps 4–6 (*Collection of colon luminal content for BMV- isolation*).
      ***Note:*** A volume of 500 μL is the maximum volume that can be loaded on each qEVoriginal / 70 nm Gen 2 column for the isolation of BMVs. Use as many filtrate devices as required to concentrate each sample (troubleshooting 1).
    iii. Cover the filtrate devices with the concentrate collection tubes by pushing the collection tubes onto the filtrate devices.
    iv. Place the filter devices into the centrifuge rotor as described above.
    v. Centrifuge at 4,000 ×*g* and 4°C for 60 min.
    vi. Remove the filter devices from the centrifuge.
    vii. Disconnect the filter devices from the filtrate collection tubes.
    viii. Then invert the filter devices tied to the concentrate collection tubes. To recover the concentrated serum and colon fluid, place the inverted filter devices in the centrifuge rotor and counterbalance with similar devices if required.
    ix. Immediately centrifuge at 1,000 ×*g* and 4°C for 2 min. This results in the transfer of the concentrated serum or colon fluid from the devices to the concentrate collection tubes.
    x. Remove the concentrate collection tubes from the devices (Figure 2).
    xi. Transfer the concentrated samples from each filter device into a 1.5 mL low protein binding collection tube. Hold the concentrated samples on ice.

### Isolation of bacterial membrane vesicles by size-exclusion chromatography

**Timing: 2 h 30 min**

Membrane vesicles (MVs) are small vesicles released by eukaryotes and prokaryotes. Bacterial membrane vesicles have diameters of 20–250 nm. Exosomes, membrane vesicles of eukaryotic origin, have a size range of 30–100 nm.^2^ On the other hand, most microvesicles mainly derived from outward budding of the plasma membrane range in sizes from 100 to 500 nm.^3^

This step describes the isolation of MVs from pre-concentrated serum and colon fluid using size-exclusion chromatography. The qEVoriginal / 70 nm Gen 2 columns were originally designed for the isolation of exosomes with a size range of 70 nm–1000 nm.^4^^,^^5^^,^^6^^,^^7^^,^^8^ However, because BMVs co-elute with exosomes, these columns can also permit the isolation of bacterial membrane vesicles (BMVs) from different biological samples.^1^

A sample volume of 500 μL is optimal for the dimension and resin volume of these columns. The qEVoriginal columns allow MVs to be separated from proteins in biological fluid because of their propensity to elute at different times in different fractions. Each fraction is defined as 0.5 mL eluate. While fractions 1–6 are considered as void volume, MVs elute in fraction 7–9, followed by the majority of proteins (>99%) in fraction 10–12.

**Figure 3:**
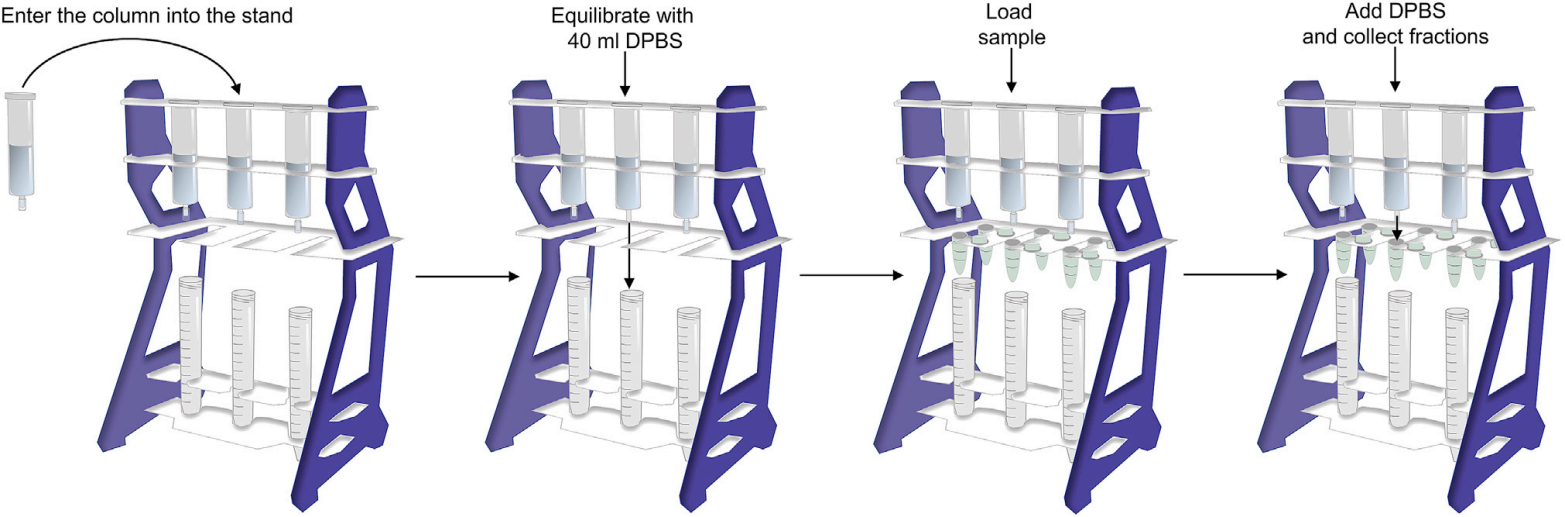
Schematic illustration of vesicle isolation from serum and colon fluid using a qEVoriginal column using a stand

8. To prepare qEVoriginal / 70 nm Gen 2 columns, the following steps need to be performed.
  ***Note:*** During BMVs isolation, a clean, sterile environment with as little as possible nucleic acid contamination is required. Include a PBS-only sample to control for any possible contamination during MV isolation via size-exclusion chromatography.
  a. Let the qEVoriginal / 70 nm Gen 2 columns and the sterile filtered DPBS reach working temperature. The operational temperature ranges from 18°C–24°C. Here, we operate at 20°C (RT).
  b. Assemble the column stand provided by IZON (Figure 3).
  c. Insert qEVoriginal / 70 nm Gen 2 columns into the stand.
  d. Insert 15 mL reaction tubes into the holder below the columns.
  e. Equilibrate each column with at least 40 mL of sterile-filtered DPBS as follows:
    i. Open the upper cap of a column and add 2 mL sterile-filtered DPBS to the filter on top of the column resin (top-filter).
    ii. Remove the luer slip cap and collect the flow-through in the 15 mL reaction tube below the column.
    iii. Constantly add DPBS on the columns, but not more than 2 mL at a time until each column is equilibrated with at least 40 mL DPBS. Whenever required, empty the 15 mL reaction tube for collecting the flow-through.
    iv. When a column is equilibrated, add the luer slip cap to close the column from the bottom to avoid loss of buffer volume.
      ***Note:*** Do not let the top-filter of the column run dry. It should be covered with DPBS.
    v. Close the upper cap of the column if the column is not immediately used.
9. To load the equilibrated qEVoriginal / 70 nm Gen 2 column with the concentrated serum or colon fluid proceed as follows.
  a. Open the upper cap of a column.
  b. Remove the remaining buffer above the top-filter while leaving the luer slip cap on the qEVoriginal column.
  c. Immediately load 500 μL of concentrated serum or colon fluid onto the center of a filter.
  d. Remove the luer slip cap and instantly start collecting 0.5 mL fractions in 1.5 mL low protein binding collection tubes.
    ***Note:*** The first 6 fractions contain the void volume but no membrane vesicles. Therefore, it is recommended to collect the void volume in two 1.5 mL collection tubes to save time and avoid measurement errors of 6 individual tubes.
  e. Add DPBS on the top-filter of the column as the last of the sample just levels the filter.
    ***Note:*** Prevent any unintentional dilution of the serum or colon fluid sample by adding DPBS before the sample enters the top-filter. Do not let the filter run dry and do not add more than 2 mL DPBS on the top-filter.
  f. Collect fraction 7 to 9 separately containing the highest amount of MVs. Store at 4°C.
  g. Collect further fractions (up to 12) that are supposed to contain higher protein content and low membrane vesicles (troubleshooting 2 and 3).
  h. After collecting the vesicle fractions, wash the column with at least 20–30 mL DPBS and store at 4°C. The top-filter should be covered with DPBS before closing the upper cap and the luer slip cap.
    ***Optional:*** Columns can be reused up to 5 times. For this, the flow rate should be tightly observed. Reduced flow rate indicates that the column was not successfully recovered for reuse.
    ***Optional:*** Measure each fraction for membrane vesicle concentration using an Izon qNano and protein purity. The protein concentration of fractions can be determined by usage of Pierce BCA protein assay kit.

### Concentration of membrane vesicle fractions

**Timing: 2 h**

This step describes the volume reduction of collected fractions containing membrane vesicles. This step was performed to increase the concentration of bacterial membrane vesicles. In our original study, we conducted 16S rDNA sequencing of amplified vesicle isolated DNA to assess the presence of gut bacteria-derived membrane vesicles in colon lumen or in blood. For this, we pooled and concentrated the MV-containing fractions to increase the bacterial DNA yield from membrane vesicles.

10. To concentrate the collected MV fractions to a final volume of 200 μL, use Amicon Ultra-2 centrifugal filter devices for volumes up to 2 mL with a cutoff of 3 kDa (see Figures 1 and 2).
  a. Equilibrate three Amicon Ultra-2 centrifugal filter devices as described in step 7a. In brief, perform the following steps:
    ***Note:*** An additional filter device should be used to concentrate the PBS-only sample that serves as negative control.
    i. Place the Amicon Ultra-2 filtrate devices into the filtrate collection tubes.
    ii. Load the filtrate devices with 500 μL ice-cold, sterile filtered DPBS and cover with the concentrate collection tubes.
    iii. Centrifuge at 4,000 ×*g* and 4°C for 60 min.
    iv. Discard the flow-through in the collection tubes, then seal the filter devices again in the collection tubes.
  b. Concentrate the MV fractions using the equilibrated Amicon Ultra-2 centrifugal filter devices as described in step 7c. In brief, perform the following steps:
    i. Pool MV fractions 6–9 from each individual sample in a 1.5 mL low protein binding collection tube and invert the tube 3-times. The volume of each MV-containing sample yields ∼1.5 mL.
    ii. Load each filtrate device with 1.5 mL MV-containing sample and cover with a concentrate collection tube.
    iii. Centrifuge at 4,000 ×*g* and 4°C for 40 min.
    iv. Remove the filter devices from the centrifuge.
    v. Disconnect the filter devices from the filtrate collection tubes.
    vi. Invert the filter devices tied to the concentrate collection tubes. Insert the inverted filter devices in the centrifuge rotor, counterbalance if required, and immediately centrifuge at 1,000 ×*g* and 4°C for 2 min.
    vii. Remove the concentrate collection tubes from the devices. Collect the concentrated serum and colon MV-containing samples with a volume of 200 μL from the concentrate collection tubes in sterile low protein binding collection tubes, then store samples at 4°C or long-term at −20°C.
      ***Optional:*** To deplete extravesicular nucleic acids from MV preparations, supplement the MV concentrate with 2 mM MgCl_2_ and 0.1 mM CaCl_2_. Then, incubate sample with 25 U/mL benzonase (Novagen) for 30 min at 37°C. Finally, add 1 mM EDTA, and heat sample at 95°C for 5 min to inhibit benzonase activity.^1^ This step is required, if aimed at 16S rDNA profiling of vesicle-encased DNA.
      ***Optional:*** Different downstream application can be performed including isolation of MV-encased DNA followed by 16S rDNA sequencing, proteomic analyses, or electron microscopy.

## Expected outcomes

This protocol allows the isolation of bacterial membrane vesicles together with exosomes from body fluids such as serum and gastrointestinal luminal content. Applying this method to isolate BMVs, we were able to extract DNA encased by bacterial membrane vesicles. Size-exclusion chromatography is an important step to separate vesicles from bulk proteins for subsequent analyses. This BMV isolation procedure paves the way for further downstream analyses e.g., electron microscopic, *in situ* hybridization, biochemical or proteomic approaches to define the composition and abundance of BMVs in different body fluids.

In our original study,^1^ we demonstrated that membrane vesicles from different bacterial phyla are present not only in the lumen of the colon but also in blood circulation. To achieve this, we purified DNA from colon and serum-isolated vesicles using AMPure XP beads (Beckman Coulter) according to the manufacturer’s instructions and then used 200 ng DNA for a two-step PCR amplification with primer sets targeting different hypervariable regions of the bacterial 16S rDNA. The PCR product was selected by appropriate size and purified for library preparation. The 16S rDNA library was analyzed by quantitative real-time PCR and by sequencing on NovaSeq 6000 SP flowcell with PE250. Finally, the 16S rDNA profile of DNA from colon and serum-isolated vesicles was compared. For more information, please see the original study.^1^ Detailed information about 16S rDNA library generation and sequencing can be found elsewhere.

## Limitations

This protocol does not distinguish between eukaryotic and bacterial vesicles. Because of the high abundance of host exosomes in body fluids such as serum, bacterial membrane vesicles only make up a very small fraction of vesicles isolated from such fluids. In combination with 16S rDNA-seq, this protocol allows the detection of microbial MVs in gastrointestinal and blood samples. To distinguish host from microbial MVs additional steps such as *in situ* hybridization or immunolabeling in combination with electron microscopy would be required.

## Troubleshooting

### Problem 1

It might be difficult to concentrate the serum to a final volume of 500 μL using Amicon Ultra-2 centrifugal filter device.

### Potential solution

Serum is very viscous due to high protein content. Therefore, concentration of serum with 3 kDa cut-off columns takes time and the membrane of the filter column might get clogged. If the volume of 500 μL cannot be reached during 1 h-centrifugation, extend the centrifugation time by 30 min. Do not increase the centrifugation force.

To prevent clogging of the filter unit membrane, reduce possible traces of blood cells/clot in serum by minimizing erythrocyte lysis during blood collection by gentle pipetting and use pipette tips with a large opening.

### Problem 2

The buffer composition and pH may affect MV stability and membrane polarity. Temperatures below 18°C or above 24°C affect qEVoriginal column function with effects on the MV elution.

The buffer system for the elution of the MVs might also not be suitable for downstream application.

### Potential solution

Instead of DPBS, other buffer systems might be used. Other buffers such as 20 mM Tris pH 7.4–8.0 for MV elution might help to stabilize BMVs. This would include that the qEVoriginal column would be equilibrated with 20 mM Tris instead of DPBS. Choose the appropriate buffer system according to your downstream application.

Due to the buffer system used, the elution of MVs might be influenced. If required, measure the MV and protein amount of the different eluted fractions.

### Problem 3

The preparation contains a mixture of BMVs and eukaryotic vesicles.

### Potential solution

To improve the recovery of bacterial membrane vesicles, qEVoriginal / 35 nm Gen 2 columns with an optimal recovery range of 35 nm–350 nm could be used to avoid losing the smaller vesicles in size range of 35–70 nm and to eliminate microvesicles larger than 350 nm.

## Resource availability

### Lead contact

Further information and requests for resources and reagents should be directed to the lead contact, Nelson O. Gekara (nelson.gekara@su.se; nelson.ongondo.gekara@uniklinik-freiburg.de).

### Materials availability

This study did not generate new unique reagents.

## Data Availability

This published article did not generate new datasets. The 16S rDNA-seq datasets generated when this protocol was optimized are available in original study^1^ and can be accessed from the NCBI Sequence Read Archive under the Bioproject 812359 identifier.
